# Inhibition of ventral tegmental area projections to the nucleus accumbens shell increases premature responding in the five-choice serial reaction time task in rats

**DOI:** 10.1007/s00429-023-02618-x

**Published:** 2023-02-27

**Authors:** Jacques P. Flores-Dourojeanni, Marleen H. van den Munkhof, Mieneke C. M. Luijendijk, Louk J. M. J. Vanderschuren, Roger A. H. Adan

**Affiliations:** 1 Department of Translational Neuroscience, Brain Center, University Medical Center Utrecht, 3508 GA Utrecht, The Netherlands; 2 Department of Population Health Sciences, Animals in Science and Society, Faculty of Veterinary Medicine, Utrecht University, 3584 CM Utrecht, The Netherlands; 3 Institute of Neuroscience and Physiology, The Sahlgrenska Academy at the University of Gothenburg, 405 30 Gothenburg, Sweden

**Keywords:** Impulsivity, Optogenetics, Halorhodopsin, 5-CSRTT, Ventral tegmental area, Nucleus accumbens shell

## Abstract

Exaggerated impulsivity and attentional impairments are hallmarks of certain disorders of behavioural control such as attention-deficit/hyperactivity disorder (ADHD), schizophrenia and addiction. Pharmacological studies have implicated elevated dopamine (DA) levels in the nucleus accumbens shell (NAcbS) in impulsive actions. The NAcbS receives its DA input from the ventral tegmental area (VTA), and we have previously shown that optogenetic activation of VTA-NAcbS projections impaired impulse control and attention in the five-choice serial reaction time task (5-CSRTT) in rats. To better understand the role of VTA-NAcbS projections in impulsivity and attention, the present study sought to inhibit this projection using optogenetics. We demonstrate that inhibiting VTA-NAcbS efferents during the last seconds of the inter-trial interval (i.e. immediately before presentation of the instructive cue) induces exaggerated impulsive action, in the absence of changes in attentional or motivational parameters in the 5-CSRTT. Together with our earlier observations, this suggests that impulse control in the 5-CSRTT is tightly controlled by VTA-NAcbS activity, with deviations in both directions resulting in increased impulsivity.

## Introduction

The ability to regulate impulses, withhold from inappropriate behaviour and maintain attention is crucial for adaptive, goal-directed behaviour. Deficits in impulse control and attentional performance can seriously impair daily functioning, and as such they feature in the symptomatology of certain mental disorders, amongst which are attention-deficit/hyperactivity disorder (ADHD), substance addiction and schizophrenia (Moeller et al. 2001).

The five-choice serial reaction time task (5-CSRTT; Carli et al., 1983) is widely used to study the neural and chemical substrates underlying impulse control and attention in rats (Bari et al. 2008). In the 5-CSRTT, rats are trained to correctly await and identify a light cue that is briefly presented in one of five nose-poke holes to obtain a food reward. Successful performance in this paradigm requires response inhibition and sustained visuospatial attention. Impulsive action is assessed by measuring premature responses (i.e. responding before the onset of the stimulus cue), whereas incorrect responses are a measure of impaired attention and response omissions are indicative of reduced attention and/or motivation. The cross-species translational significance of the 5-CSRTT is highlighted by comparable studies performed in both rats and humans; the latter using human analogues of the abovementioned rodent 5-CSRTT (Voon et al. 2014; Worbe et al. 2014).

Dopamine (DA) is well known to be involved in the modulation of impulsive and attentional behaviours (Dalley and Roiser 2012; Nieoullon 2002; Pattij and Vanderschuren 2008). Indeed, drugs that increase brain DA transmission, such as amphetamine and methylphenidate, are used to normalize impulsivity and attention in the treatment of ADHD (Sharma and Couture 2014). Yet, the specific brain mechanisms through which DA acts to regulate impulse control and attention are incompletely understood. Previous studies have shown that impulsive and attentional parameters of 5-CSRTT performance are regulated by distinct DAergic cortico-striatal circuits. Incorrect responses and omissions are mostly impacted by DA manipulations in the medial prefrontal cortex (mPFC) and dorsal striatum (Agnoli et al. 2013; Baunez and Robbins 1999), whereas DA manipulations in the ventral striatum have a profound effect on premature responding (Cole and Robbins 1989). Thus, impulsive behaviour is generally exacerbated by drugs that enhance DA transmission in the NAcb, such as amphetamine and methylphenidate (Dalley and Robbins 2017; Economidou et al. 2012; Pattij and Vanderschuren 2008; Robbins 2018).

However, accumulating evidence indicates that the association between elevated DA transmission in the NAcb and impaired impulse control in the 5-CSRTT is not as straightforward as previously assumed. Importantly, the NAcb is not a functionally homogenic structure. It comprises two functionally distinct areas, i.e. the core (NAcbC) and shell (NAcbS) subregions (Zaborszky et al. 1985; Zahm and Brog 1992), and it has been suggested that DA acts through opponent actions in the NAcbC and NAcbS to modulate impulsivity in the 5-CSRTT. For instance, the DA D2/3 receptor antagonist nafadotride significantly increases premature responding when infused into the NAcbS region, but decreases premature responding when infused into the NAcbC of highly impulsive rats (Besson et al. 2010). Moreover, lesions to the NAcbC potentiate amphetamine induced increased impulsivity, whereas lesions to the shell attenuate this effect (Murphy et al. 2008).

Although it is well known that the NAcb receives its DA input from the VTA, previous studies have mostly focused on local mechanisms within the NAcb. Remarkably, Boekhoudt et al. (2017) did not find any effects on premature responses when chemogenetically stimulating the VTA. However, that study did not distinguish between NAcbC and the NAcbS. Whereas, by specifically targeting VTA projections towards the NAcbS with time-locked precision, we found a pronounced increase in the number of premature responses (Flores-Dourojeanni et al. 2021). In this study, we sought to optogenetically silence NAcbS projecting VTA neurons in rats to examine their role in both impulsive action and attention. Given our previous results, we hypothesize that inhibiting this projection will result in decreased impulsivity and increased attentional performance in the 5-CSRTT. However, the effects of nafadotride suggest inhibition of the NAcbS may increase premature responses.

## Materials and methods

### Animals

A total of ten house-bred Long Evans male rats were used in this experiment. Female rats were excluded to avoid potential behavioural changes incurred by their estrous cycle (Verharen et al. 2019). The rats were socially housed in Macrolon type IV cages until surgery, after which animals were housed individually in Macrolon III cages to prevent damage to the implanted chronic fibres. All cages contained saw dust as bedding and a wood block was provided for cage enrichment. To enable behavioural testing during the animals’ active phase, the animals were housed under a reversed 12-h day–night cycle (lights off at 07:00) in a temperature- and humidity-controlled room (20–21 °C; 60–70%). Throughout behavioural training and testing, the rats were food restricted to 15 g of chow per day (Special Diet Services, Essex, UK), to maintain around 90% of their free-feeding body weight. At all times, the animals had ad libitum access to water in their home cage. All the experiments were approved by the Animal Ethics Committee of Utrecht University and were conducted in accordance with the European Directive (2016/63/EU).

### Stereotaxic surgery

Anaesthesia was induced by an intramuscular injection of fentanyl/fluanisone (0.315 mg/kg fentanyl, 10 mg/kg fluanisone, Hypnorm, Janssen Pharmaceutica) and were subsequently placed in a stereotaxic apparatus (Kopf Instruments). Xylocaine (Lidocaine 100 mg/mL, AstraZeneca BV) was sprayed on the skull as a local analgesic. To obtain projection-specific targeting of VTA projections towards the NAcbS, we combined a retrograde canine adeno-associated virus delivering Cre-recombinase (Cav2-Cre; Montpellier Vector Core) with halorhodopsin (NpHR). All the animals were bilaterally injected with 0.4 μL of Cav2-Cre (1.0 × 10^12^ molecules/mL) into the NAcbS at AP: + 1.20, ML: + 2.70, DV: − 7.50 under a 10° angle. The animals also received a bilateral injection of 1.0 μL AAV5-hsyn-DIO-NpHR3.0-eYFP (1.0 × 10^12^ molecules/mL; UNC vector Core) into the VTA at AP: − 5.60, ML: + 1.30, DV: − 8.20 under a 5° angle. Lastly, chronic optic fibres (127–131 μm core diameter, 0.22 NA, Precision Fiber Products) were placed into the VTA slightly above the injection site (DV: − 7.70) and fixed to the skull with self-adhesive cement (RelyX^™^ Unicem Aplicap kit, 3 M, USA). All viruses were injected at a rate of 0.2 μL/min, where after the needle was left in place for 10 min to maximize diffusion. After surgery, animals received a subcutaneous injection of 1.0 mL saline to prevent dehydration and a second subcutaneous injection of 1.0 mL/kg Carprofen (5.0 mg/kg, Carporal, AST Farma BV) for pain relief. Carprofen was also given daily for the two following days. During recovery, rats had ad libitum access to food and water, and they were housed individually. There was a minimum of 9 weeks between surgery and behavioural testing.

### 5-CSRTT apparatus and training

Training and testing in the 5-CSRTT took place in operant conditioning chambers (30.5 × 24 × 21 cm, Med-Associates, Georgia, USA). The chambers were equipped with five horizontally spaced nose-poke holes in a curved wall, each of which contained a yellow light emitting diode (LED) stimulus light. In the opposite wall, a food magazine for sucrose pellet (45 mg, TestDiet, USA) delivery was located. The nose-poke holes and food magazine contained an infrared detector to enable the measuring of nose-pokes or head entries. A houselight** was placed above the food magazine and the floor consisted of a metal grid with saw dust below. The operant conditioning chambers were placed in sound attenuated boxes, which were equipped with a fan to provide ventilation and a low level of white noise. All inputs and outputs of the chambers were controlled by MED-PC version 4.2 (Med-Associates).

After a short period of habituation to the chamber, the rats were trained over different stages (see Van Gaalen et al 2006; Baarendse and Vanderschuren 2012; Boekhoudt et al. 2017) until reaching the typical 5-CSRTT baseline. During such baseline conditions, the inter-trial interval (ITI) is set at 5 s, the stimulus light cue at 1 s, the limited hold at 5 s, and the time-out at 5 s. Training sessions in the standard 5-CSRTT took place daily. Training sessions consisted of 100 trials or 30 min, whichever occurred first. Every session started with the delivery of one free pellet, after which the first trial was initiated by a head entry into the food magazine. Each trial thereafter started with a 5 s ITI, followed by a 1 s stimulus light cue that was presented in a pseudo-random order in one of the five nose-poke apertures. A correct response was counted when a rat responded into the illuminated hole within the limited hold of 5 s, which was immediately rewarded with a sucrose pellet. A nose-poke made during the ITI was recorded as a premature response and a nose-poke during the limited hold into the wrong hole was recorded as an incorrect response. When the animal failed to respond within the limited hold, this was recorded as an omission. Perseverant responses were recorded as nose-pokes made into any hole aperture after a correct response but prior to reward retrieval. Premature responses, incorrect responses and omissions were punished with a 5 s time-out, during which the stimulus light and the houselight were both switched off. The next trial started either automatically after this time-out or, in case of a previous correct response, when the animal made a head entry into the food magazine. All responses and their latencies were recorded with Med-Associates apparatus. Optical testing started after approximately 4 months, when the animals showed stable performance under baseline 5-CSRTT conditions.

### Optogenetic set-up and experimental procedure

Optogenetic manipulations were performed as follows. Lasers (Changchun New Industries, Optoelectronics TECH. CO.) were attached to a rotary joint (Doric Lenses, Quebec, CA) via a mono fibre-optic patch cord (200 μm core diameter, 0.22 NA, Doric Lenses). The rotary joint was in turn connected to two patch cords (made in-house, 127–131 μm core diameter, 0.22 NA, THORLABS), which were bilaterally attached to the implanted chronic fibres of an animal located in the operant chamber. The animals were able to move freely whilst being attached to the patch cords. Laser light with a wavelength of 532 nm was used for optogenetic inhibition. Lasers were continuously on during inhibition and laser intensity was maintained between 10 and 15 mW, which was measured before and after every test session. To minimize light leakage around the attachment of the patch cords to the chronic optic fibres, the cement cap was coloured black and black tape was wrapped around the attachment site during testing. To control for any possible side effects of the remaining light pollution, mock stimulation sessions were performed. These mock sessions were identical to test sessions, but the patch chords were blocked such that no light entered the animals’ brains.

**Fig. 1:**
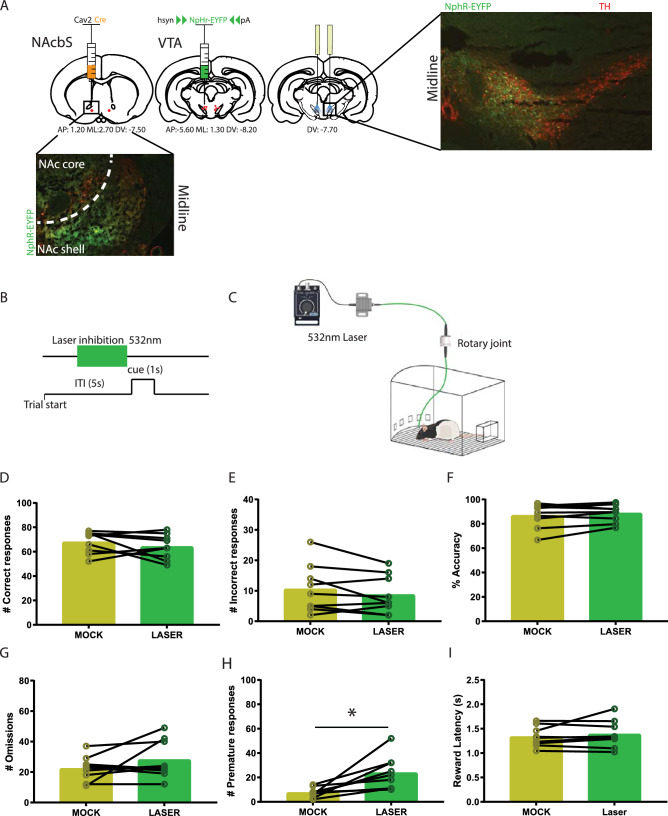
Inhibition of VTA projections towards the NAcbS in the 5-CSRTT. **A** Infusion of the Cre-dependent AAV5-hsyn-DIO-NpHR3.0-eYFP viral vector into the VTA; TH (red) and NpHR3-EYFP (green). **B** Schematic of the 5-CSRTT depicting the time point of inhibition in green. Inhibition took place in the last 3 s of the inter-trial interval (ITI). **C** Schematic of the in vivo optogenetics set-up. **D**–**I**, correct responses, incorrect responses, accuracy, omissions, premature responses and latency to retrieve the reward during mock and laser stimulation sessions, respectively (*n* = 9). Data are presented as mean ± SEM; *P < 0.025 when compared to mock stimulation (Paired sample Student’s *t* test)

**Fig. 2:**
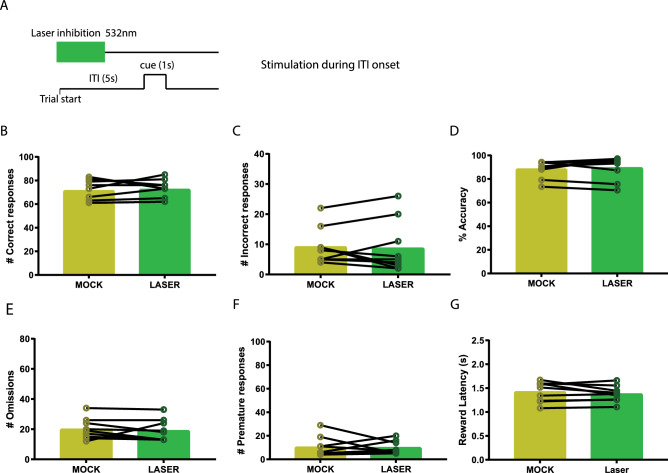
Inhibition of VTA projections towards the NAcbS in the 5-CSRTT at the onset of the ITI. **A** Schematic of the 5-CSRTT depicting time point of inhibition in green. Inhibition took place in the first 3 s of the inter-trial interval (ITI). **B**–**G**, Correct responses, incorrect responses, accuracy, omissions, premature responses, and latency to retrieve the reward during mock and laser stimulation sessions, respectively (*n* = 9). Data are presented as mean ± SEM

**Fig. 3:**
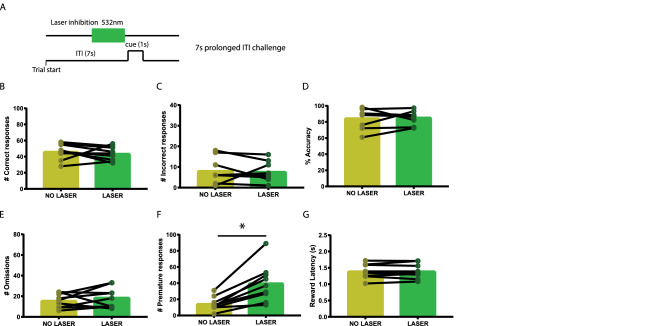
Inhibition of VTA projections towards the NAcbS in the 5-CSRTT during a 7 s extended ITI challenge. **A** Schematic of the 5-CSRTT depicting time point of inhibition in green. Inhibition took place in the last 3 s of the inter-trial interval (ITI). **B**–**G**; Correct responses, incorrect responses, accuracy, omissions, premature responses, and latency to retrieve the reward during no-laser and laser trials within a session, respectively (*n* = 9). Data are presented as mean ± SEM; *P < 0.025 when compared to mock stimulation (Paired sample Student’s *t* test)

**Fig. 4:**
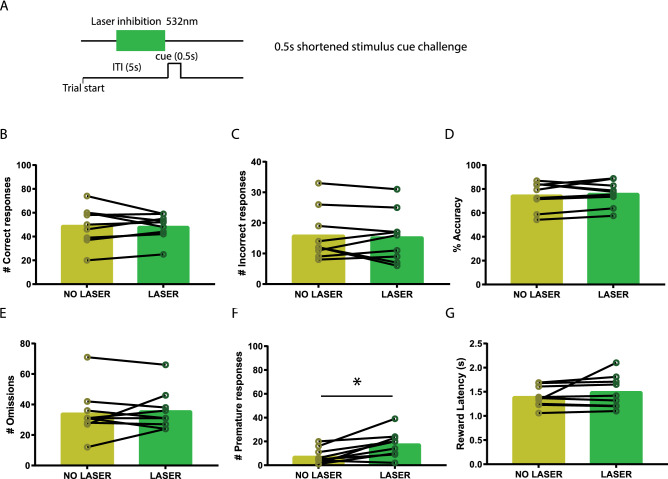
Inhibition of VTA projections towards the NAcbS in the 5-CSRTT during a 0.5 s shortened stimulus cue challenge. **A** Schematic of the 5-CSRTT depicting time point of inhibition in green. Inhibition took place in the last 3 s of the inter-trial interval (ITI). **B**–**G**, Correct responses, incorrect responses, accuracy, omissions, premature responses, and latency to retrieve the reward during no-laser and laser trials within a session (respectively, *n* = 9). Data are presented as mean ± SEM; *P < 0.025 when compared to mock stimulation (Paired sample student’s *t* test)

To examine at what time during 5-CSRTT performance VTA to NAcbS signalling is most important, time-locked optogenetic manipulation was applied at two different time frames in a standard 5-CSRTT trial: for 3 s prior to cue presentation (Fig. 1B) and for 3 s at the onset of the ITI (Fig. 2A). Test sessions in the standard 5-CSRTT consisted of 100 laser trials and were identical to the training sessions apart from the laser stimulation. Moreover, the effect of our optogenetic manipulation was tested in two distinct 5-CSRTT challenges. In these challenges, ITI and cue parameters slightly deviated from the baseline conditions in which the animals had been trained, to assess the animals’ capability to cope with increased cognitive demands and changes in task contingencies. Here, we tested an extended ITI challenge, in which the ITI was extended from 5 to 7 s (Fig. 3A) and a shortened cue challenge (Fig. 4A), in which the cue was shortened from 1 to 0.5 s. To prevent habituation bias to the altered parameters in these challenges, challenge sessions were only executed once for every animal. Every challenge test session consisted of blocks of 10 laser trials that were alternated with blocks of 10 no-laser trials for a total duration of 200 trials. In each laser trial, the laser was switched on for a duration of 3 s prior to cue presentation. Lastly, animals only underwent optogenetic stimulation if they had shown stable performance (< 10% variation in accuracy and omissions) in the 2 days prior to optogenetic stimulation.

### Tissue preparation and immunofluorescence

Upon completion of the experiments, the rats received a lethal dose of sodium pentobarbital (0.2 mL/100 g bodyweight i.p., Euthanimal, Alfasan BV, The Netherlands). After extinction of their withdrawal reflex, the animals were transcardially perfused with 0.01 M phosphate-buffered saline (PBS) followed by 4% paraformaldehyde (PFA) in PBS. The brains were removed and post-fixed in PFA at 4˚ for a minimum of 24 h, after which they were transferred to a 30% sucrose solution for at least 2 days before being sliced into 40 μm coronal sections (cryostat, Leica CM 1950). The brain sections were stored in phosphate buffer (PB) with 0.05% sodium azide up until staining for tyrosine hydroxylase (TH) and eYFP with Mouse anti-TH (EMD Millipore, 1:500) and Chicken anti-eYFP (Sigma-Aldrich, 1:1000), respectively. The sections were incubated overnight at 4˚ with these antibodies, where after they were incubated for 2 h with the secondary antibodies Goat anti-Mouse 488 (Molecular Probes, 1:500) and Goat anti-Chicken 568 (Molecular Probes, 1:500). All antibodies were dissolved in a buffer solution, containing 92% PB, 5% Triton, and 3% Normal Goat Serum (NGS). Afterwards, the sections were mounted using FluorSave (EMD Millipore) and examined with a fluorescence microscope (AxioCam MRm, Zeiss). Brain sections were checked for expression of halorhodopsin (NpHR) in the VTA as well as in the NAcbS (Fig. 1A).

### Data analysis

No animals were excluded from the behavioural analysis based on NpHR expression. However, one animal lost its chronic implants before testing, leaving a total of nine rats for the analysis. Behavioural results are presented as means (± SEM) for each variable (correct response, premature response, incorrect response, omission, accuracy, latency to reward collection, correct latency, and premature response latency). Accuracy was defined as the percentage of correct responses relative to the total amount of responses (correct / [correct + incorrect] × 100%). Other parameters were analyzed as absolute values. Laser inhibition sessions during baseline 5 s ITI sessions were compared to mock sessions. Whilst in the challenge test sessions, the laser trials were compared to no-laser trials within the same session. The differences between laser and mock/no-laser session/trials were tested using a paired Student’s *t *test, after checking for normal distribution with a Shapiro–Wilk test. Statistical significance was set at *P* < 0.05. Statistical tests and graphs were made using GraphPad Prism 6.

## Results

### Inhibition of VTA projections towards the NAcbS increases premature responding

In the present study, we sought to inhibit the projection from the VTA to the NAcbS by optogenetic inhibition using halorhodopsin (NpHr). We found that inhibition of VTA efferents towards the NAcbS 3 s prior to cue presentation did not induce changes in the number of correct (Fig. 1D; *t* = 1.1, *P* = 0.31) or incorrect responses (Fig. 1E; *t* = 1.5, *P* = 0.16), accuracy (Fig. 1F; *t* = 1.21, *P* = 0.26) or omissions (Fig. 1G; *t* = 1.5, *P* = 0.18). Mock laser stimulation (consisting of blocked light) did not impact on task performance. In a previous study using a similar set-up, we used YFP controls to ensure that laser stimulation itself did not disrupt behaviour (Flores-Dourojeanni et al. 2021). In contrast, it greatly increased the number of premature responses (Fig. 1H; *t* = 3.5, *P* = 0.009). This effect was present in the absence of changes in reward collection (Fig. 1I; *t* = 0.97, *P* = 0.36), correct response (Table 1; *t* = 0.96, *P* = 0.36), or premature response latencies (Table 1; *t* = 0.36, *P* = 0.72) or in the number of perseverative responses (Table 1; *t* = 0.16, *P* = 0.87).

**Table 1:** The impact of varying inter-trial intervals (ITI) on performance in the 5-CSRTT

	Perserverative responses		Latency			
			Correct		Premature	
	no-laser	laser	no-laser	laser	no-laser	laser
5 s ITI,	3.2 ± 0.7	1.8 ± 1.1	0.62 ± 0.04	0.65 ± 0.05	3.6 ± 0.29	3.5 ± 0.16
5 s ITI, ITI onset stimulation	3.6 ± 0.9	3.2 ± 1.0	0.68 ± 0.06	0.66 ± 0.06	3.7 ± 0.12	3.7 ± 0.14
7 s ITI	2.0 ± 0.6	2.5 ± 0.8	0.65 ± 0.06	0.69 ± 0.05	5.5 ± 0.22	5.2 ± 0.15
5sITI, 0.5 s cue	2.3 ± 0.9	2.1 ± 1.2	0.56 ± 0.05	0.63 ± 0.1	3.8 ± 0.16	3.5 ± 0.13*

### Premature responses are only increased when VTA-NAcbS inhibition occurs at the end of ITI period

To assess whether the effects of optogenetic VTA-NAcbS inhibition were restricted to a specific time point during the ITI, we inhibited these neurons at the onset of the ITI rather than at the end. Our results showed no changes in the number of correct (Fig. 2B; *t* = 0.52, *P* = 0.61) or incorrect responses (Fig. 2C; *t* = 0.30, *P* = 0.77), accuracy (Fig. 2D; *t* = 0.59, *P* = 0.57) or omissions (Fig. 2E; *t* = 0.56, *P* = 0.59). In addition, there was no increase in the number of premature responses (Fig. 2F; *t* = 0.14, *P* = 0.88). There were also no effects on reward collection (Fig. 2G; *t* = 1.1, *P* = 0.29), correct response (Table 1; *t* = 0.64, *P* = 0.54) or premature response latencies (Table 1; *t* = 0.14, *P* = 0.88), or in the number of perseverative responses (Table 1; *t* = 0.35, *P* = 0.73).

### The effects of inhibiting of VTA-NAcbS projections on premature responding remain during task challenges

Increasing the ITI from 5 to 7 s is known to increase premature responses in rats (Dalley et al., 2007; Baarendse and Vanderschuren 2012; Boekhoudt et al. 2017). We next tested the effects of inhibiting VTA projections to the NAcbS during this long ITI challenge. We found no effects of VTA-NAcbS inhibition on the number of correct (Fig. 3B; *t* = 0.71, *P* = 0.49) or incorrect (Fig. 3C; *t* = 0.28, *P* = 0.78) responses, accuracy (Fig. 3D; *t* = 0.28, *P* = 0.78) or omissions (Fig. 3E; *t* = 0.98, *P* = 0.35). Premature responses (Fig. 3F; *t* = 4.7, *P* = 0.0014) were significantly increased in laser trials, this increase was stronger than during the 5 s ITI session (mean of differences ± SEM of differences for 5sITI = 16.2 ± 4.7; and for 7 s ITI challenge = 25.6 ± 5.4). There was no effect on the latencies to collect the reward (Fig. 3G; *t* = 0.05, *P* = 0.95), or the latencies to correct (Table 1; *t* = 0.77, *P* = 0.46) and premature (Table 1; *t* = 1.57, *P* = 0.15) responses. Perseverative (Table 1; *t* = 0.41, *P* = 0.69) responses were also not affected by inhibition.

Last, we sought to test whether inhibiting VTA to NAcbS projections could alter attention or impulsivity in the 5-CSRTT when the attentional demand was increased, by reducing the duration of the stimulus cue (Baarendse and Vanderschuren 2012; Boekhoudt et al. 2017). We found no changes in correct (Fig. 4B; *t* = 0.27, *P* = 0.79) or incorrect (Fig. 4C; *t* = 0.45, *P* = 0.66) responses, accuracy (Fig. 4D; *t* = 0.75, *P* = 0.47) or omissions (Fig. 4E; *t* = 0.52, *P* = 0.61) between laser and no-laser trials. Premature (Fig. 4F; *t* = 3.72, *P* = 0.0056) responses were significantly increased by VTA-NAcbS inhibition; however, this increase was smaller than that observed during the 5sITI session and the 7sITI challenge (mean of differences ± SEM of differences for 0.5 short stimulus challenge = 8.2 ± 2.7). There was no effect on reward collection latency (Fig. 4G; *t* = 1.2, *P* = 0.27), correct response latency (Table 1; *t* = 1.23, *P* = 0.25) or perseverative responses (Table 1; *t* = 0.40, *P* = 0.69). However, there was a significant decrease in premature response latency (Table 1; *t* = 3.63, *P* = 0.0067) of optogenetic inhibition of the VTA-NAcbS projection.

**Fig. 5:**
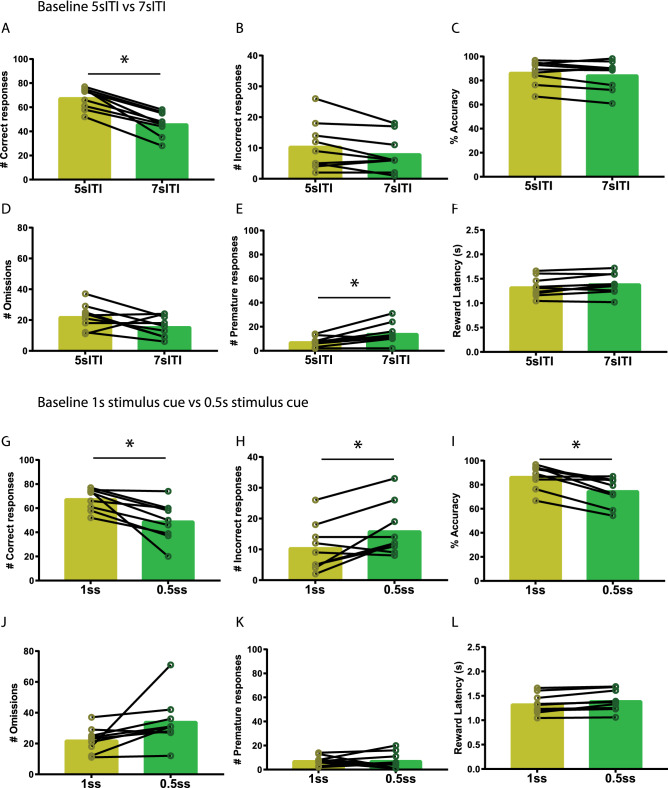
Comparing baseline 5-CSRTT (5 s ITI and 1 s stimulus cue) with 5-CSRTT 7sITI and 0.5 s stimulus cue challenges. **A–F** Correct responses, incorrect responses, accuracy, omissions, premature responses, and latency to retrieve the reward during baseline 5sITI (yellow) and 7sITI challenge (green), respectively (*n* = 9). Data are presented as mean ± SEM. **G**–**L** Correct responses, incorrect responses, accuracy, omissions, premature responses, and latency to retrieve the reward during baseline 1 s stimulus cue (yellow) and 0.5 s stimulus cue challenge (green), respectively (*n* = 9). * *P* < 0.025 when compared to mock stimulation (Paired sample Student’s *t* test)

Increasing the ITI from 5 to 7 s effectively increased premature responses. To assess whether the 7sITI challenge effectively increased the number of premature responses in the animals, we compared no-laser 7sITI challenge trials with baseline 5-CSRTT 5sITI mock trials. We found correct responses were significantly decreased (Fig. 5A; *t* = 8.02, *P* < 0.0001). Incorrect responses (Fig. 5B; *t* = 2.23, *P* = 0.056), accuracy (Fig. 5C; *t* = 1.53, *P* = 0.16) and omissions (Fig. 5D; *t* = 2.02, *P* = 0.08)) were not affected but premature responses (Fig. 5E; *t* = 3.14, *P* = 0.014) increased as predicted. The latency to retrieve the reward increased (Fig. 5F; *t* = 3.4, *P* = 0.01), but the effect size was small (mean of differences ± SEM of differences = 0.06 ± 0.02).

Decreasing the stimulus cue from 1 s to 0.5 s effectively decreased incorrect responses and accuracy.

We also compared the no-laser trials in the 0.5 s shortened cue challenge with baseline 5-CSRTT 1 s stimulus cue mock trials. We found a decrease in correct responses (Fig. 5G; *t* = 3.71, *P* = 0.006), an increase in incorrect responses (Fig. 5H; *t* = 2.83, *P* = 0.02) and a decrease in accuracy (Fig. 5I; *t* = 4.47, *P* = 0.002). Omissions (Fig. 5J; *t* = 2.20, *P* = 0.06), premature responses(Fig. 5K; *t* = 0.05, *P* = 0.96), and the latency to retrieve the reward(Fig. 5L; *t* = 2.25, *P* > 0.05) remained unaffected. The latency to retrieve the reward is marginally significant but the effect size was small (mean of differences ± SEM of differences = 0.08 ± 0.03).

## Discussion

In this study, we used time-locked optogenetic inhibition to silence VTA projections towards the NAcbS during 5-CSRTT performance in rats. We found that reducing activity of these projections resulted in exaggerated premature responding. This effect was only present when inhibition took place near the end of the ITI, highlighting the temporal importance of this projection. Furthermore, these effects were selective for premature responding since they did not affect attentional or motivational performance. Demonstrating that inhibition of VTA inputs to the NAcbS, in a timing-specific manner, increases impulsive action.

Earlier studies have provided substantial evidence for the involvement of DAergic transmission in the NAcbS in impulsivity in the 5-CSRTT (Besson et al 2010; Dalley and Robbins 2017; Economidou et al 2012; Moreno et al., 2013; Murphy et al 2008; Pezze et al 2007). Pharmacological studies have demonstrated that premature responding is associated with high levels of DA in this region (Dalley and Robbins 2017; Diergaarde et al. 2008; Robbins 2018; Murphy et al 2008; Jupp et al 2013), which is in line with the exacerbating effect of VTA-NAcbS activation on premature responses we found previously (Flores-Dourojeanni et al. 2021).

It is important to emphasize that halorhodopsin has been previously known to induce a post-inhibitory excitation of neurons upon termination of laser stimulation (Mattingly et al. 2018). This is thought to be a result of a lowered threshold of firing caused by alterations in the ionic gradient. However, the effects observed on premature responses occurred within the period of inhibition (Mattingly et al. 2018). Since the post-inhibitory effects of halorhodopsin occur after inhibition (after the laser has been turned off), we can safely discount this as an alternative explanation of our results. Nevertheless, the relationship between DA and impulsivity remains unclear, as demonstrated by our previous studies wherein stimulating VTA DA neurons did not increase premature responding (Flores-Dourojeanni et al. 2021; Boekhoudt et al. 2017). The complexity of DA’s role in impulse control may be a result of the different DA receptor subtypes within the NAcb. The principal cell types in the NAcb are GABAergic medium spiny neurons (MSNs), which can be categorized into two functionally distinct populations based on their expression of either the D1 or D2/3-type DA receptor. Pharmacological studies have found differential effects of DA D1 and D2/3 receptor inactivation on 5-CSRTT performance. Local infusion of a DA D1 receptor antagonist into the NAcbS of rats reduced impulsivity on the 5-CSRTT (Pattij and Vanderschuren 2008), whereas infusions of a DA D2/3 receptor antagonist increased impulsive premature responding (Besson et al. 2010). Furthermore, trait-like impulsivity in rats is linked to decreased DA D2/3 receptor availability in the NAcbS (Besson et al. 2010; Dalley and Robbins 2017; Jupp et al. 2013). Oral administration of methylphenidate has been found to alleviate this exaggerated premature responding in highly impulsive rats, whilst also upregulating DA D2/3 receptor availability in the ventral striatum (Caprioli et al. 2015). This suggest that optogenetic inhibition of NAcbS projecting VTA neurons causes an increase in premature responding by decreasing the activity of DA D2/3 receptors. Moreover, it is possible that the increase in premature responding previously observed during optogenetic stimulation may be driven by changes in D1 receptor signalling. Although, this remains to be tested. Put together, our findings imply that impulse control in the 5-CSRTT requires a balance of DA levels in the NAcbS during the critical time point preceding the cue. Disrupting this balance by changing VTA to NAcbS signalling induces exaggerated impulsive premature responding.

Previously we found that chemogenetic activation of VTA DA neurons increases the number of omissions (Boekhoudt et al. 2017). Similarly, we also showed that activating VTA efferents towards the NAcbS or the NAcbC provide the same effect (Flores-Dourojeanni et al. 2021). In this study, however, we show that inhibition of VTA-NAcbS projections does not alter the number of omissions. To date, the role of DA transmission on errors of omission in the 5-CSRTT is not entirely clear. Region-specific studies that have investigated the role of D1 and D2/3 DA receptors in the NAcbS have found that infusion of both D1 and D2/3 antagonists increases the number of omissions in the 5-CSRTT (Pattij et al. 2007; Besson et al. 2010; Pezze et al. 2007). In contrast, inactivation of the NAcbS via infusions of the GABA agonist muscimol does not alter the number of omissions (Feja et al. 2014). The lack of attentional effects in this experiment may indicate that impulse control is more sensitive to fluctuations in NAcb DA than attention. After all, optogenetic silencing is partial (not all projecting neurons will express NpHr) and time-locked (restricted to a portion of the ITI). These aspects can allow for enough neuronal signalling to pass through and enable proper attentional behaviour. Alternatively, VTA projections to the NAcbC may compensate for the inhibition of NAcbS efferents since both regions have been shown to play a role in attentive behaviour.

To specifically target VTA neuron projections towards the NAcbS, we used the Cav2-Cre retrograde tracer and injected it into nucleus accumbens shell coordinates. We are aware that Cav2-Cre may fail to infect all the different subsets of neurons. Future experiments may opt for more efficient retrograde tracers to increase the number of projections cells targeted (Li et al. 2018). Additionally all rats showed Halo staining on axon terminals in the shell of the accumbens but there will was inevitably some spread to surrounding areas which varied between rats. Our optogenetic manipulations targeted VTA neurons that innervate the NAcbS. Although this projection also comprises non-DA neurons (Morales and Margolis 2017; Yamaguchi et al. 2011), it is well-established that over 80% of the VTA projections to the NAcb emerge from DA neurons (Swanson 1982). Consistently, previous studies in our lab, using the same two-viral approach in this pathway, have found a similar proportion of infected VTA-NAcb neurons to be DAergic (Boender et al. 2014; Verharen et al. 2018). Moreover, using in-vivo microdialysis, we have shown increased baseline levels of DA and its metabolites in the NAcb after chemogenetic activation of this projection (Verharen et al. 2018). Nevertheless, a contribution from GABAergic and glutamatergic neurons may still play an important role in mediating the observed behaviours. For instance, muscimol infusions into the NAcbS has been shown to increase premature responses in the 5-CSRTT (Feja et al. 2014). However, GABA infusions in that study also greatly increased all response latencies whereas we found an increase in premature responses in the absence of any latency changes. This may indicate that our manipulation is driven by a different mechanism. Also, inhibition of GABAergic neurons in the VTA would cause a disinhibition of neurons in the NAcbS which would contrast the effects of a GABA agonist in the NAcbS. However, since we have demonstrated that both inhibition and stimulation of VTA-NAcbS efferents disrupt impulse control, the idea that a fine balance of stimulation and inhibition is necessary to maintain proper impulse control, may still hold true for either DA or GABAergic transmission. Concerning glutamatergic neurons, optogenetic inhibition of VTA Vglut2 + neurons projecting to the NAcb has been shown to prevent foot-shock-driven increased immobility in a forced swim test (Qi et al. 2016). These results suggest glutamatergic neurons in the VTA may play a role in behavioural inhibition within the NAcb. Altogether this study complements our previous findings showcasing the importance of VTA projections towards the NAcbS. By inhibiting cells with time-locked precision during the last seconds of the ITI of the 5-CSRTT, we were able to increase premature responding without altering any other parameters. We show, together with our previous findings, that a fine balance of activity emerging from the VTA towards the NAcbS is essential for maintaining proper inhibitory control.

## Data Availability

The datasets generated during and/or analyzed during the current study are available in the DataverseNL Utrecht University repository; Replication Data for: Inhibition of ventral tegmental area projections to the nucleus accumbens shell increases premature responding in the 5-Choice serial reaction time task in rats.
